# Abstract Spatial Reasoning as an Autistic Strength

**DOI:** 10.1371/journal.pone.0059329

**Published:** 2013-03-22

**Authors:** Jennifer L. Stevenson, Morton Ann Gernsbacher

**Affiliations:** 1 Department of Psychology, Ursinus College, Collegeville, Pennsylvania, United States of America; 2 Department of Psychology, University of Wisconsin–Madison, Madison, Wisconsin, United States of America; Ecole Normale Supérieure, France

## Abstract

Autistic individuals typically excel on spatial tests that measure abstract reasoning, such as the Block Design subtest on intelligence test batteries and the Raven’s Progressive Matrices nonverbal test of intelligence. Such well-replicated findings suggest that abstract spatial processing is a relative and perhaps absolute strength of autistic individuals. However, previous studies have not systematically varied reasoning level – concrete vs. abstract – and test domain – spatial vs. numerical vs. verbal, which the current study did. Autistic participants (N = 72) and non-autistic participants (N = 72) completed a battery of 12 tests that varied by reasoning level (concrete vs. abstract) and domain (spatial vs. numerical vs. verbal). Autistic participants outperformed non-autistic participants on abstract spatial tests. Non-autistic participants did not outperform autistic participants on any of the three domains (spatial, numerical, and verbal) or at either of the two reasoning levels (concrete and abstract), suggesting similarity in abilities between autistic and non-autistic individuals, with abstract spatial reasoning as an autistic strength.

## Introduction

Enhanced spatial perception is a signature characteristic of autistic individuals (e.g., [1]; see Sinclair’s (1999) essay, “Why I dislike person first language” [51], for why we have chosen to use the term “autistic person(s)” rather than “person(s) with autism”). The most replicated autistic strength is on the embedded figures task: Autistic individuals locate hidden figures in a visual display more rapidly than non-autistic individuals do [2], [3], [4], [5], [6]. In fact, the speed with which autistic participants correctly locate hidden figures distinguishes them from non-autistic participants more powerfully (*d* = 2.8) than do measures of theory of mind (*d* = 1.0) or executive function (*d* = 0.3–1.1) [7]. Even non-autistic adults who have more autistic traits than other non-autistic adults locate hidden figures more rapidly and accurately [8].

Strengths can be identified in either absolute or relative terms. A relative strength, or a personal strength, is an area in which an individual excels compared with other areas in which the individual performs less well. An absolute strength, such as a population strength, is an area in which a population or group of individuals excel, compared with another group of individuals who perform less well. Autistic individuals’ strength on the embedded figures test is a highly replicated absolute strength.

A highly replicated relative strength for many autistic individuals is their performance on Block Design subtests, which occur on various intelligence tests [9], [10], [11]. To complete a Block Design subtest, the participant arranges small blocks to replicate a target pattern. Although some studies report Block Design performance as an autistic absolute strength (i.e., autistic individuals perform Block Design better than non-autistic individuals [7], [12], [13]), and some non-autistic individuals who have more autistic traits perform Block Design better than other non-autistic individuals who have fewer autistic traits [14], [15], most studies report Block Design as a relative autistic strength (i.e., autistic individuals perform Block Design subtests better than other subtests).

In fact, autistic individuals usually show a marked peak on the Block Design subtest [16], [17], [18], [19], [20]. Table 1 summarizes the results of nearly 40 studies of autistic participants’ performance on Wechsler intelligence test batteries. On average, autistic participants perform two-thirds of a standard deviation higher on the Block Design subtest than they do on other subtests illustrating a relative autistic strength.

**Table 1:** Studies Reporting Wechsler Subtest Scores for Autistic Children and Adults.

First Author, Year [Citation]	*N*	*M*	Range	Best Subtest	Worst Subtest
Allen, 1991 [16]	20	5.28	9.90	Block Design	Comprehension
Asarnow, 1987 [52]	23	8.71	8.30	Block Design	Comprehension
Bartak, 1975 [53]	9	6.90	9.70	Block Design	Comprehension
Bölte, 2002 [54]	20	7.69	3.80	Object Assembly	Picture Arrangement
Bölte, 2004 [55]	59	7.11	2.94	Similarities	Picture Arrangement
Charman, 2011 [29]	127	6.33	3.70	Picture Arrangement	Comprehension
Dawson, 2011 [31]	57	10.02	4.92	Information	Coding/Digit Symbol
de Bruin, 2006 [56]	100	8.54	2.34	Picture Arrangement	Coding/Digit Symbol
Dennis, 1999 [57]	8	9.26	4.37	Block Design	Comprehension
Ehlers, 1997 [58]	80	8.70	3.60	Similarities	Coding/Digit Symbol
Freeman, 1985 [59]	21	9.07	5.60	Block Design	Comprehension
Gilchrist, 2001 [60]	33	7.96	4.23	Block Design	Coding/Digit Symbol
Goldstein, 2002 [61]	31	9.34	6.16	Information	Coding/Digit Symbol
Happé, 1994 [17]	51	4.67	4.33	Block Design	Comprehension
Holdnack, 2011 [62]	43	8.26	3.21	Similarities	Coding/Digit Symbol
Koyama, 2006 [63]	27	9.44	5.50	Block Design	Comprehension
Koyama, 2007 [64]	73	9.73	4.17	Digit Span	Comprehension
Koyama, 2008 [18]	106	10.32	3.00	Digit Span	Comprehension
Koyama, 2009 [65]	142	9.65	3.37	Block Design	Comprehension
Lincoln, 1988 [66]	46	6.01	7.40	Block Design	Comprehension
Lockyer, 1970 [67]	varies	5.09	5.44	Block Design	Coding/Digit Symbol
Mayes, 2003 [68]	63	9.22	1.83	Similarities	Coding/Digit Symbol
Mayes, 2004 [69]	93	10.25	4.70	Similarities	Coding/Digit Symbol
Mayes, 2008 [70]	54	10.05	6.30	Similarities	Coding/Digit Symbol
Merchán-Naranjo, 2011 [71]	29	9.47	5.83	Information	Coding/Digit Symbol
Minshew, 2005 [72]	215	9.66	3.61	Information	Coding/Digit Symbol
Narita, 1987 [73]	45	5.23	8.98	Block Design	Comprehension
Noterdaeme, 2010 [74]	112	9.79	4.05	Information	Picture Arrangement
Nyden, 2001 [75]	20	10.55	7.00	Vocabulary	Digit Span
Ohta, 1987 [76]	16	5.85	9.20	Block Design	Comprehension
Rumsey, 1988 [77]	10	10.55	7.65	Block Design	Comprehension
Shah, 1988^a^ [17]	18	5.94	9.60	Block Design	Comprehension
Siegel, 1996 [19]	81	8.98	3.07	Block Design	Coding/Digit Symbol
Spek, 2008 [78]	43	11.44	2.72	Comprehension	Coding/Digit Symbol
Szatmari, 1990 [24]	43	7.77	2.18	Block Design	Comprehension
Tymchuk, 1977 [79]	20	8.16	4.37	Digit Span	Comprehension
Venter, 1992 [20]	52	6.41	4.35	Block Design	Comprehension
Williams, 2006 [80]	38	10.50	5.60	Information	Comprehension
Average (weighted equally)	38	8.36	4.02	Block Design	Comprehension
Average (weighted by *N*)	2028	8.74	3.05	Block Design	Comprehension

*N* = number of participants; *M* = average score; Range = range of scores.

a Unpublished data as reported in Happé (1994).

Autistic individuals’ strength on Block Design tests is often interpreted, not as an area of cognitive strength, but instead as an area of diagnostic weakness. For example, a popular theory proposes that autistic individuals excel on Block Design because they actually suffer from weak central coherence – a reduced ability to ‘see the big picture.’ According to this theory, autistic individuals excel at decomposing the target pattern because their perception is too piecemeal [13]. However, even when the typical Block Design task is reversed – participants have to match a pre-segmented target to an intact pattern – autistic participants still excel [21].

When autistic individuals’ strength on Block Design is not being re-interpreted as a weakness, it is often dismissed as reflecting merely low-level [22] or concrete processing [23], [24]. However, according to psychometricians, Block Design must draw on complex problem solving because it is so highly correlated with general intelligence [25], [26]. Tests that correlate highly with general intelligence, as Block Design does, are considered more general, and, therefore, more abstract cognitive tests. Their high correlation with general intelligence suggests that they draw on more components of general intelligence [25], [26].

In contrast to Block Design, the Coding/Digit Symbol subtest, for which autistic individuals typically do not excel, is only moderately correlated with general intelligence [25]. Tests that correlate only moderately with general intelligence are considered more specific, and, therefore, more concrete cognitive tests. Note that abstractness and concreteness, when applied to cognitive tests, are not synonymous with more and less difficult. Rather, more general, abstract tests are those that correlate more highly with general intelligence, whereas less general, concrete tests are those that correlate only moderately with general intelligence [25], [26]. Block Design is a more abstract cognitive test, because it draws on more components of intelligence, whereas Coding/Digit Symbol is considered more concrete, because it draws on fewer components of general intelligence [25], [26]. Therefore, contrary to some autism researchers’ assumptions, autistic individuals’ strength on Block Design is a strength in abstract spatial reasoning.

Block Design is not the only abstract spatial test on which autistic individuals excel. The Raven’s Progressive Matrices [27] is an even more abstract test [25], [26], and autistic individuals show a relative strength on Raven’s Progressive Matrices. Autistic children and adults score higher on Raven’s Progressive Standard Matrices than they score on Wechsler tests [28], [29], [30], [31]; in contrast, non-autistic children and adults score the same across the two types of tests. When autistic and non-autistic children are matched on their performance on Wechsler tests, autistic children are more accurate than non-autistic children on the Raven’s Standard Progressive Matrices [32]. When autistic and non-autistic adults are equal in their accuracy on the Raven’s Standard Progressive Matrices, autistic individuals are faster [33]. Even for non-autistic adults, their degree of autistic traits predicts their successful completion of Raven’s Standard Progressive Matrices items that are considered more visual-spatial [34]. Thus, autistic individuals’ performance on the Raven’s Progressive Matrices, the most agreed upon test of abstract spatial reasoning, suggests a strength in abstract spatial reasoning.

**Figure 1:**
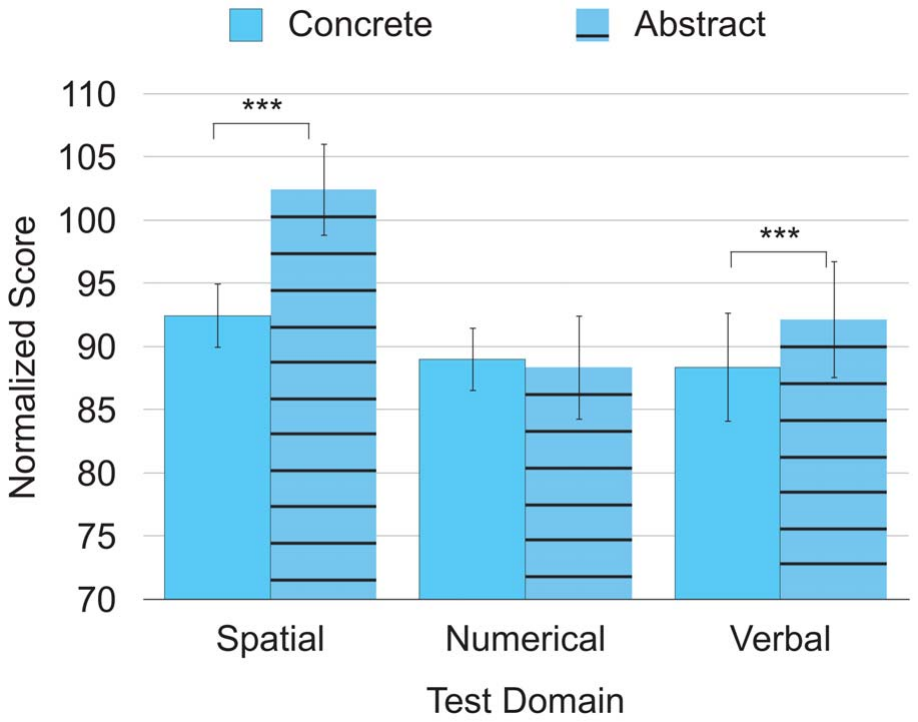
Interaction between test domain and reasoning level in previous studies. Data summarized from nearly 40 previous studies of autistic participants’ performance on Wechsler subtests. Composite scores were normalized to have a *M* = 100 and *SD* = 15. Error bars represent 2 *SE*. ***p≤.001.

Further evidence for autistic individuals’ strength in abstract spatial reasoning can be approximated from their performance profile on the Wechsler intelligence scales. The subtests on Wechsler scales vary in both reasoning level (concrete or abstract) [25], [26] and test domain (spatial, numerical, or verbal). As shown in Table 2, autistic participants perform better on Wechsler subtests that tap the spatial domain (e.g., Block Design, Picture Completion) than they perform on subtests that tap the numerical domain (e.g., Arithmetic, Digit Symbol/Coding) or verbal domain (e.g., Vocabulary, Comprehension). Autistic participants also perform better on Wechsler subtests that assess abstract reasoning (e.g., Vocabulary, Arithmetic, Block Design) than on subtests that assess concrete reasoning (e.g., Comprehension, Digit Symbol/Coding, Picture Completion). Thus, Figure 1 suggests that autistic participants have strength in abstract spatial processing.

**Table 2:** Effects of Test Domain and Reasoning Level in Wechsler Subtest Scores for Autistic Children and Adults.

First Author, Year [Citation]	Concrete Spatial	AbstractSpatial	Concrete Numerical	Abstract Numerical	Concrete Verbal	Abstract Verbal
Allen, 1991 [16]	87.58	106.00	73.38	71.75	62.38	64.13
Asarnow, 1987 [52]	98.67	117.50	87.50	91.00	88.00	86.75
Bartak, 1975 [53]	96.00	112.00	87.25	78.00	66.25	72.25
Bölte, 2002 [54]	87.00	93.00	86.50	87.50	88.50	90.75
Bölte, 2004 [55]	83.56	89.37	84.07	86.15	84.47	88.75
Charman, 2011 [29]	86.67	80.00	80.00	80.50	78.25	79.75
Dawson, 2011 [31]	97.00	100.80	85.50	102.80	101.10	107.80
de Bruin, 2006 [56]	93.68	92.90	87.70	90.75	94.05	95.73
Dennis, 1999 [57]	96.27	108.75	95.90	95.65	90.03	97.20
Ehlers, 1997 [58]	90.92	99.00	89.38	87.75	97.50	97.63
Freeman, 1985 [59]	99.17	110.50	91.25	90.50	92.25	91.75
Gilchrist, 2001 [60]	87.83	102.00	89.33	88.30	88.53	89.10
Goldstein, 2002 [61]	97.92	103.40	86.85	99.70	95.65	100.90
Happé, 1994 [17]	76.37	86.35	76.60	67.15	68.73	66.68
Holdnack, 2011 [62]	n/a	93.85	86.90	90.90	92.80	93.10
Koyama, 2006 [63]	98.67	110.00	100.75	94.50	89.25	94.25
Koyama, 2007 [64]	96.40	108.75	100.53	101.25	94.08	98.30
Koyama, 2008 [18]	100.07	108.40	103.18	100.95	97.85	102.93
Koyama, 2009 [65]	97.15	106.10	100.43	97.90	94.30	97.93
Lincoln, 1988 [66]	87.23	101.20	79.88	76.60	69.33	71.20
Lockyer, 1970 [67]	75.68	91.40	74.70	72.05	70.90	72.60
Mayes, 2003 [68]	96.50	98.50	92.42	92.17	96.75	99.33
Mayes, 2004 [69]	102.33	108.00	91.00	95.00	103.00	108.00
Mayes, 2008 [70]	n/a	108.00	87.50	50.00	105.50	107.50
Merchán-Naranjo, 2011 [71]	102.53	93.30	82.60	89.45	98.10	104.75
Minshew, 2005 [72]	95.95	104.30	94.40	101.00	96.38	103.28
Narita, 1987 [73]	73.78	102.00	91.63	68.35	59.55	71.90
Noterdaeme, 2010 [74]	93.78	104.60	89.05	102.85	99.25	106.63
Nyden, 2001 [75]	95.00	110.00	90.75	95.50	109.25	120.00
Ohta, 1987 [76]	85.33	103.00	81.75	72.50	64.50	73.75
Rumsey, 1988 [77]	98.00	124.50	104.50	102.50	96.88	103.13
Shah, 1988^a^ [17]	87.50	111.00	75.50	72.50	71.75	68.00
Siegel, 1996 [19]	94.88	101.85	91.85	95.25	92.58	96.60
Spek, 2008 [78]	105.95	110.10	101.33	108.85	109.88	110.13
Szatmari, 1990 [24]	89.45	93.60	88.03	88.40	85.88	89.53
Tymchuk, 1977 [79]	89.93	99.00	90.68	89.10	88.20	91.55
Venter, 1992 [20]	83.62	91.25	85.35	77.50	78.00	77.35
Williams, 2006 [80]	100.13	108.30	96.90	105.65	98.83	110.98
Average (weighted equally)	92.46	102.44	89.02	88.37	88.38	92.15
Average (weighted by *N*)	89.20	101.01	90.56	91.21	91.47	95.40

Concrete Spatial tests comprise Picture Completion, Picture Arrangement, and Object Assembly; Abstract Spatial comprises Block Design; Concrete Numerical comprises Digit Symbol/Coding and Digit Span; Abstract Numerical comprises Arithmetic; Concrete Verbal comprises Comprehension and Similarities; and Abstract Verbal comprises Vocabulary and Information. Composite scores were normalized to have a *M* = 100 and *SD* = 15. An ANOVA on these data indicated significant main effects of domain (*F*(2, 70) = 20.31, *p*<0.001) and reasoning level (*F*(1, 35) = 88.73, *p*<0.001) and a significant interaction between domain and reasoning level (*F*(2, 70) = 17.07, *p*<0.001).

a Unpublished data were reported in Happé (1994).

However, this analysis of Wechsler subtest performance does not systematically vary reasoning level, abstract versus concrete, and domain, spatial versus non-spatial. In fact, no previous study has systematically varied reasoning level and test domain. That was the purpose of the present study, which examined relative and absolute strengths in autistic and non-autistic individuals’ performance on 12 tests that varied by reasoning level (concrete vs. abstract) and domain (spatial vs. numerical vs. verbal).

## Methods

### Ethics Statement

Participants gave written consent, approved by the Institutional Review Board at the University of Wisconsin–Madison to ensure the study was ethical. In addition, the Gateway Council (a committee of academic researchers and autistic adults) ensured that the study was inclusive, respectful, accessible, and relevant.

### Participants

Participants were recruited through the Gateway Project (http://thegatewayproject.org), which is an Internet-based research platform for inclusive, respectful, accessible, and relevant studies involving autistic and non-autistic adults. Internet-based studies, including studies that administer cognitive tests, provide results comparable to in-person studies [35], [36]. For example, Internet-based administration of reading and math tests provide results that are highly correlated with in-person administration of the same tests on the same participants [37].

Participants in the Gateway Project complete a 30-minute enrollment survey (i.e., the Gateway Survey) that collects demographic data, such as age, personal and parental education level, and includes the Autism-Spectrum Quotient [38]. For the current study, autistic participants were defined as adults who met criteria for the autism spectrum on the Autism-Spectrum Quotient and self-identified as autistic. Non-autistic participants were adults who did not meet criteria for the autism spectrum on the Autism-Spectrum Quotient and did not self-identify as either being autistic or as having any other disability.

All participants were native English speakers who did not report being blind or having significant vision loss lasting at least 6 months. Data were analyzed from 72 autistic adults (36 males, 36 females) and 72 non-autistic adults (36 males, 36 females) who completed all 12 cognitive tests. Autistic and non-autistic participants were matched on sex, age, personal education, and parental education (all *t*(142) <1, all *p*s >.01). A summary of the participants’ demographic characteristics is provided in Table 3.

**Table 3:** Demographic Characteristics for Autistic and Non-Autistic Participants.

	Autistic	Non-Autistic	*p*
*N*	72	72	
AQ Percent: *M* (*SD*)	78.26 (9.68)	33.56 (11.09)	<.001
Formal Diagnosis: No/Yes	23/49	72/0	<.001
Age at Session 1: *M* (*SD*)	41.64 (12.49)	41.47 (12.22)	.94
Age at Session 2: *M* (*SD*)	41.68 (12.50)	41.51 (12.22)	.94
Age at Session 3: *M* (*SD*)	41.69 (12.51)	41.53 (12.22)	.94
Sex: Male/Female	36/36	36/36	1.00
Personal Education: *M* (*SD*)	15.76 (2.09)	15.67 (1.91)	.77
Parent Education: *M* (*SD*)	15.44 (2.82)	15.47 (2.89)	.95
Latino or Hispanic: No/Yes	70/2	70/2	1.00
Racial Category: White/Other	68/4	68/4	1.00
Country: US/Other	59/13	68/4	.02

### Materials

The 12 cognitive tests varied by reasoning level (concrete or abstract) and domain (spatial, numerical, or verbal). All 12 tests were time-limited, and all but the two concrete numerical tests were multiple-choice. Nine of the 12 cognitive tests were taken from the Educational Testing Service (ETS) Kit of Factor-Referenced Cognitive Tests [39], with the other three cognitive tests taken from the Cognitive Abilities Test [40]. Table 4 summarizes the reasoning level, test domain, number of items, time limit, and correlation with general intelligence for each test.

**Table 4:** Description of Cognitive Tests.

Cognitive Test	Reasoning Level	Domain	Number of Items	Time Limit (in minutes)	Correlation with General Intelligence [25], [26]
Card Rotations	Concrete	Spatial	160	6	*r = *.3
Cube Comparisons	Concrete	Spatial	42	6	*r* = .5
Paper Folding	Abstract	Spatial	20	6	*r = *.7
Figure Analogies	Abstract	Spatial	20	10	*r = *.7^a^
Addition	Concrete	Numerical	120	4	*r* = .3
Subtraction and Multiplication	Concrete	Numerical	120	4	*r* = .3
Necessary Arithmetic Operations	Abstract	Numerical	30	10	*r* = .7
Number Analogies	Abstract	Numerical	24	12	*r = *.7^a^
Vocabulary I	Concrete	Verbal	36	8	*r* = .3^a^
Extended Range Vocabulary	Concrete	Verbal	48	12	*r* = .3^a^
Letter Sets	Abstract	Verbal	30	14	*r* = .7^a^
Verbal Analogies	Abstract	Verbal	30	10	*r* = .8

a Correlation from comparable test (e.g., Pattern Analogies for Figure Analogies).

The four spatial tests were Card Rotations, Cube Comparisons, Paper Folding, and Figure Analogies. In Card Rotations, participants determine which of eight options represents a two-dimensional target when rotated or flipped. In Cube Comparisons, participants determine whether two three-dimensional cubes are the same or different, allowing for each to be rotated. In Paper Folding, participants determine which of five drawings represent how a sheet of folded paper would appear when unfolded. In Figure Analogies, participants select among five geometric figures the geometric figure that forms a pair analogous to another pair of geometric figures. Card Rotations and Cube Comparisons served as the two concrete spatial tests, while Paper Folding and Figure Analogies served as the two abstract spatial tests.

The four numerical tests were Addition, Subtraction and Multiplication, Necessary Arithmetic Operations, and Number Analogies. On the Addition test, participants add three one- or two-digit numbers (e.g., 80+78+15). On the Subtraction and Multiplication test, participants alternate between subtracting two-digit numbers from two-digit numbers (e.g., 98–75) and multiplying two-digit numbers by one-digit numbers (e.g., 86×6). On the Necessary Arithmetic Operations test, participants identify the numerical operations required to solve arithmetic word problems (e.g., If a man earns $5.75 an hour, how many hours should he work each day in order to make an average of $46.00 per day? *Subtract, Divide, Add, or Multiply*). On the Number Analogies test, participants select among five numbers the number that forms a relation analogous to two previous sets of relations between numbers (e.g., [5 → 4 ] [8 → 7 ] [3 → ? ] *1, 2, 3, 5,* or *6*). The Addition Test and the Subtraction and Multiplication Test served as the two concrete numerical tests, while Necessary Arithmetic Operations and Number Analogies served as the two abstract numerical tests.

The four verbal tests were Vocabulary I, Extended Range Vocabulary, Letter Sets, and Verbal Analogies. On the Vocabulary I and Extended Range Vocabulary tests, participants select among four words the synonym of a target word (e.g., jovial: *refreshing, scarce, thickset, wise,* or *jolly*). On Letter Sets, participants select among five sets of letters the letter set that obviates the pattern established by the other four letter sets (e.g., NOPQ, DEFL, ABCD, HIJK, or UVWX). On Verbal Analogies, participants select among five words the word that forms a relation analogous to a previous relation between two words (e.g., [cow → milk : chicken → ?] *feather, dinner, egg, hen,* or *bird*). Vocabulary I and Extended Range Vocabulary served as the two concrete verbal tests, while Letter Sets and Verbal Analogies served as the two abstract verbal tests.

### Procedure

Participants, who met eligibility requirements, were notified of the study via email. They were administered the 12 cognitive tests over three 45-minute sessions. Each session assessed a single test domain (i.e., spatial, numerical, or verbal, in that order). During each session, participants completed two concrete tests followed by two abstract tests. As is standard practice, the tests were administered in the same order for all participants. At the conclusion of each session, participants were able to enter a drawing with a 1 in 10 chance of receiving a $25 gift card. Participants who completed all three sessions received an additional $10 gift card.

Each session began with a set of unrelated warm-up items, which is a procedure demonstrated to reduce participant drop-out in Internet-based studies [41], [42]. Before each test, participants read detailed instructions and completed practice items. At the conclusion of each session, participants stated whether they performed the tests to the best of their ability.

### Data Analysis

A conservative α = .01 was used for all analyses because of the multiple comparisons conducted in this study. A raw score for each participant on each test was computed according to scoring criteria provided by the ETS Factor-Referenced Cognitive Tests and the Cognitive Abilities Test. A participant’s raw score was excluded from further analysis if the participant took more than 10 more seconds beyond the time limit for that test; the participant failed to respond correctly to any item on that test; or if there was high accuracy in a very short time on that test. Less than 5% of the raw scores for each cognitive test were excluded (*M* = 2.12%, *SD* = 0.86, range = 0.95%–4.36%), and the likelihood of exclusion did not differ between autistic and non-autistic participants (all χ^2^s >0.05 and <3.0, all *p*s >.01).

Before further analysis, the raw score distributions for all 12 tests were inspected for normality (kurtosis and skewness within ±2). The raw scores for 2 of the tests, Letter Sets and Verbal Analogies, were cube-transformed because their distributions were leptokurtic, indicating a higher probability of extreme scores. The distribution of Verbal Analogies raw scores was also negatively skewed. After cube-transformation, the raw score distributions for Letter Sets and Verbal Analogies were relatively normal (Letter Sets before cube-transformation: kurtosis = 2.18; Letter Sets after cube-transformation: kurtosis = −0.24; Verbal Analogies before cube-transformation: kurtosis = 7.35 and skew = −2.51; Verbal Analogies after cube-transformation: kurtois = 0.68 and skew = −0.85).

Participants’ raw scores fell within 1 *SD* of a sample of 11^th^ and 12^th^ graders, on Cube Comparisons, Paper Folding, and Letter Sets, and within 2 *SD* on Extended Range Vocabulary [39]. Furthermore, Spearman-Brown split-half correlations between odd and even items on each test ranged from *r*(70) = .81 to *r*(70) = .98 in autistic participants and *r*(70) = .75 to *r*(70) = .99 in non-autistic participants, suggesting good internal consistency.

For comparisons among tests, raw scores for each test were normalized to have a *M* = 100 and *SD* = 15 because standard scores are not provided for the ETS Factor-Referenced Cognitive Tests. For comparisons among domains and reasoning levels, six composite scores, concrete spatial, abstract spatial, concrete numerical, abstract numerical, concrete verbal, and abstract verbal, were computed by averaging participants’ normalized scores from each composite’s two source tests.

## Results

Participants’ raw and normalized scores on the 12 tests are presented in Table 5, and all raw data are available by request from the first author. A group (2) × domain (3) × reasoning level (2) mixed-design ANOVA indicated a significant three-way interaction (*F*(2, 284) = 8.99, *p*<.001). This three-way interaction was also significant when the sample was expanded to all participants who completed the study, 103 autistic and 148 non-autistic, although this larger sample was unmatched for sex, age, personal education, and parent education (*F*(2, 498) = 16.79, *p*<.001). The three-way interaction between group, domain, and reasoning level was also significant when the sample was reduced to only those of autistic participants with a formal diagnosis (49 autistic; 72 non-autistic; *F*(2, 238) = 11.39, *p*<.001). No other interactions or main effects were significant (all *p*s >.01).

**Table 5:** Means (and Standard Deviations) for Raw and Normalized Test Scores.

	Raw Test Scores		Normalized Test Scores		
Cognitive Test	Autistic	Non-Autistic	Autistic	Non-Autistic	*p*
Card Rotations (Concrete – Spatial)	93.38 (25.79)	104.57 (26.69)	96.86 (14.46)	103.14 (14.97)	.01
Cube Comparisons (Concrete – Spatial)	21.03 (9.76)	18.57 (8.94)	101.96 (15.56)	98.04 (14.25)	.12
Paper Folding (Abstract – Spatial)	12.90 (4.01)	10.52 (3.55)	104.50 (15.19)	95.50 (13.46)	<.001
Figure Analogies (Abstract – Spatial)	14.82 (4.38)	13.60 (3.94)	102.18 (15.67)	97.82 (14.07)	.08
Addition (Concrete – Numerical)	21.69 (8.41)	24.68 (7.74)	97.27 (15.62)	102.73 (14.18)	.03
Subtraction and Multiplication (Concrete – Numerical)	33.88 (12.55)	36.33 (11.49)	98.47 (15.40)	101.53 (14.30)	.22
Necessary Arithmetic Operations (Abstract – Numerical)	18.72 (5.89)	18.64 (4.57)	100.12 (16.82)	99.88 (13.04)	.93
Number Analogies (Abstract – Numerical)	13.31 (5.16)	13.04 (4.57)	100.41 (15.94)	99.59 (14.10)	.75
Vocabulary I (Concrete – Verbal)	31.33 (5.01)	30.46 (4.26)	101.40 (16.14)	98.60 (13.74)	.26
Extended Range Vocabulary (Concrete – Verbal)	33.35 (8.90)	30.98 (7.73)	102.12 (15.90)	97.88 (13.83)	.09
Letter Sets^a^ (Abstract – Verbal)	21.12 (4.98)	21.13 (5.17)	99.83 (14.83)	100.17 (15.27)	.89
Verbal Analogies^a^ (Abstract – Verbal)	25.42 (3.36)	25.40 (3.46)	100.05 (15.64)	99.95 (14.44)	.97

a *t*-test conducted on cubed transformed raw scores.

**Figure 2:**
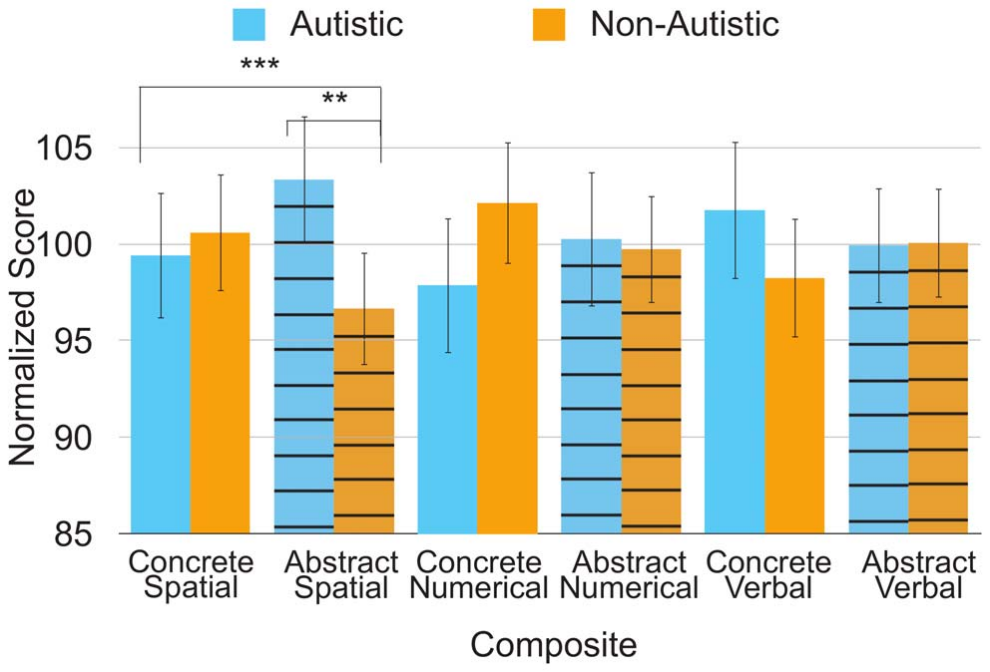
Interaction of group and reasoning level at domain. Solid colors represent concrete tests and horizontal stripes represent abstract tests. Error bars represent 2 *SE*. **p≤.01, ***p≤.001.

To explore further the significant three-way interaction between group, test domain, and reasoning level, post-hoc domain x reasoning level repeated-measures ANOVAs were conducted within each group to assess relative strengths, and post-hoc group x reasoning level mixed-design ANOVAs were conducted across the three domains to assess absolute strengths. Domain and reasoning level significantly interacted for both autistic (*F*(2, 142) = 4.60, *p* = .01) and non-autistic participants (*F*(2, 142) = 4.40, *p* = .01). As shown in Figure 2, autistic participants exhibited a relative advantage for the abstract reasoning composite in the spatial domain (*F*(1, 71) = 12.64, *p* = .001). Non-autistic participants exhibited the reverse pattern of performance with a relative advantage for the concrete reasoning composite in the spatial domain (*F*(1, 71) = 8.88, *p* = .004). Reasoning level did not significantly affect performance in the numerical or verbal domains for either autistic (numerical: *F*(1, 71) = 2.28, *p* = .14; verbal: *F*(1, 71) = 1.28, *p* = .26) or non-autistic participants (numerical: *F*(1, 71) = 1.74, *p* = .19; verbal: (*F*(1, 71) = 1.49, *p* = .23). Furthermore, the interaction between group and reasoning level was significant in the spatial domain (*F*(1, 142) = 20.86, *p*<.001), with autistic participants exhibiting an absolute strength over non-autistic participants on abstract spatial tests (*F*(1, 142) = 9.11, *p* = .003), but not on concrete spatial tests (*F*(1, 142) = 0.28, *p* = .60).

**Figure 3:**
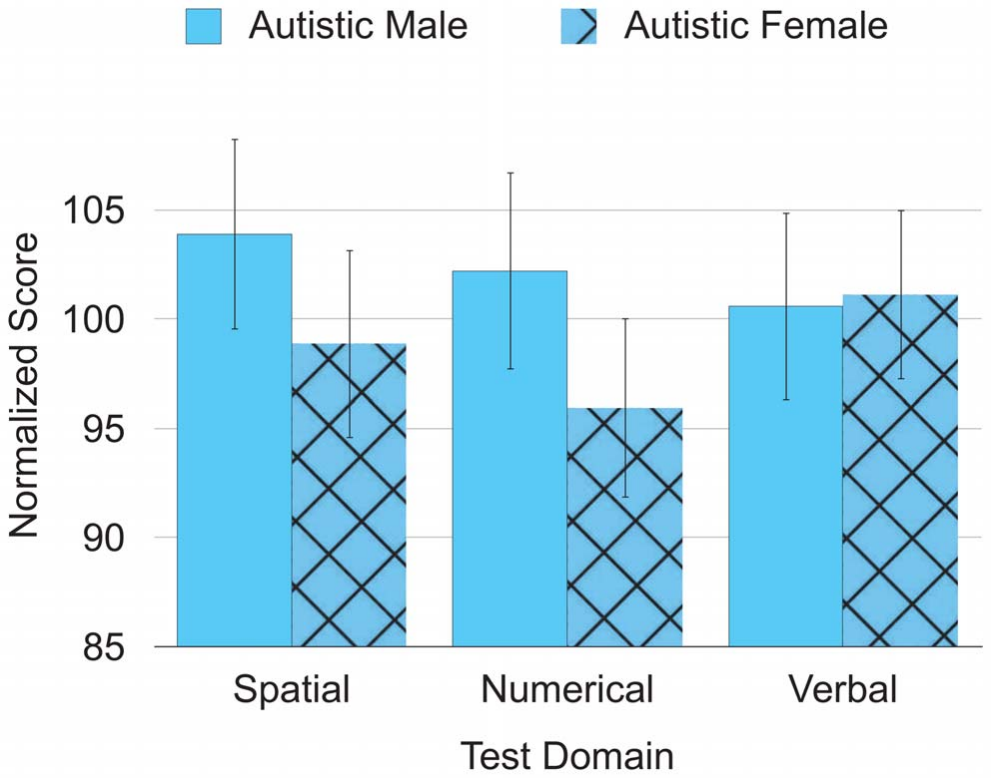
Interaction of sex and domain for autistic participants. Error bars represent 2 *SE*.

A sex × group × domain × reasoning level mixed-design ANOVA examined sex differences in performance on the cognitive tests. Sex did not interact significantly with either domain (*F*(2, 280) = 3.03, *p* = .05) or group (autistic vs. non-autistic, *F*(1, 140) = 2.95, *p* = .09). Figure 3 shows performance of the autistic group broken down by sex.

## Discussion

The present study varied reasoning level (concrete and abstract) and test domain (spatial, numerical, and verbal) to examine more systematically previous suggestions of a relative and absolute autistic strength on tests of abstract spatial reasoning. Autistic participants performed significantly better on abstract spatial tests than concrete spatial tests, suggesting spatial abstract reasoning is a relative autistic strength. Furthermore, autistic participants performed significantly better than non-autistic participants on abstract spatial tests, suggesting that spatial abstract reasoning is also an absolute autistic strength. Autistic participants’ superior performance on the abstract spatial tests used in this study rebuffs the assumption that their previously documented strengths on Block Design [17] and Raven’s Progressive Matrices [30] arise from rote memory or low-level concrete processing or that autistic individuals are impaired on tests that require abstract reasoning [43], [44].

From where does autistics’ abstract spatial strength arise? We don’t know, but we do know that very early in life autistic children are considerably more receptive to abstract spatial stimuli than are non-autistic children. In a recent study, autistic toddlers split their time evenly examining abstract spatial stimuli (on one side of a computer monitor) and videos of unknown children (on the other side of the monitor) [45]. Typically developing toddlers, and toddlers with other developmental disabilities, glanced only occasionally at the abstract spatial stimuli. Only a few non-autistic toddlers split their viewing time equally between the abstract spatial stimuli and the videos of unknown children, despite the abstract spatial stimuli extending across half the computer monitor placed before them. Later in life, many autistic adults also express a preference for spatial representations compared with verbal representations [46].

With regard to the verbal domain, in the current study, autistic participants performed as well as the non-autistic participants on both concrete verbal tests and abstract verbal tests. Such a finding might appear to contradict the well-established weakness autistic participants display on some of the Wechsler subtests that tap the verbal domain, most notably the Wechsler Comprehension subtest. However, the verbal tests in the present study differ from the verbal Wechsler subtests in several ways. The verbal tests in the present study offered multiple-choice responses to written stimuli, whereas the Wechsler verbal subtests require overt language production to spoken questions. The verbal subtests on the Wechsler scales also require face-to-face interaction with an administrator, whereas all the tests in the present study avoid this potential confound.

The data in present study resemble the pattern of sex differences in cognitive abilities sometimes found in other studies, with a slight, but not always significant, male advantage on the spatial [47] and numerical domains [48] and a slight, but not always significant, female advantage on the verbal domain [49]. In the present study, this pattern of slight, but not significant, sex differences occurred for both autistic and non-autistic participants. Such a finding seems at odds with a recently popularized theory about autism: the extreme male brain theory [50], which claims that male and female autistic individuals not only mirror but exaggerate non-autistic male strengths (i.e., an advantage on spatial and numerical tests and a disadvantage on verbal tests). However, autistic females resembled the pattern usually found with non-autistic females, and autistic males resembled the pattern usually found with non-autistic males. Thus, the present results argue against autistic strengths as simply an extreme version of male strengths.

The present study is the first large-scale Internet-based examination of autistic and non-autistic cognition. Internet-based research platforms minimize the social and communication barriers often present in more traditional laboratory settings. The current study’s success supports the use of the Internet as a research medium for investigating other cognitive domains. Most importantly, the results further our understanding of the nature of autistic cognition and its strengths.
